# Green food choices under mortality salience: a dual-path mechanism of self-esteem and cultural worldview

**DOI:** 10.3389/fnut.2026.1726176

**Published:** 2026-07-01

**Authors:** Weihuan Su, Xiaodong Guo

**Affiliations:** 1 Faculty of Business Administration, Shanxi University of Finance and Economics, Taiyuan, China; 2 School of Management, Wuhan University of Technology, Wuhan, China

**Keywords:** approach-avoidance motivation, culture worldview, green food, mortality salience, perceived competence, perceived warmth, self-esteem

## Abstract

**Background:**

Climate crisis, pandemics, and food safety scandals have exacerbated public survival anxiety, driving a shift in consumer priorities toward safety and sustainability, propelling green food into the market mainstream.

**Methods:**

Grounded in Terror Management Theory, this study constructed a dual-path framework to examine the effect of mortality salience on consumers’ green food purchase intention. Four between-subjects controlled experiments (*N* = 913) were conducted, with ANOVA, Bootstrap mediation and moderated mediation analysis to test the hypotheses.

**Results:**

The results demonstrate that mortality salience concurrently activates two distinct psychological pathways: an approach motivation, driven by a cultural worldview aligned with pro-social and environmental values, and an avoidance motivation, rooted in self-esteem and linked to economic considerations of personal and familial health protection. Furthermore, consumer perceptions of green food selectively moderate these pathways: perceived warmth facilitates the social-culturally oriented approach route, whereas perceived competence strengthens the economically-concerned avoidance route.

**Conclusion:**

These findings under-score the necessity of integrating both socio-cultural appeals and economic-functionality considerations into strategies aimed at promoting green consumption.

## 1 Introduction

Currently, human health faces a multi-faceted assault from climate upheaval, recurring pandemics, and food safety scandals. Firstly, extreme heat persistently shatters records, while torrential floods and severe droughts occur within the same season. Concurrently, rising sea levels and thawing permafrost risk releasing ancient pathogens, systematically eroding the fundamental foundations of human health (1). Secondly, the virus epidemic is evolving into a persistent “new normal.” From COVID-19 to chikungunya fever (CHIKV), pathogens are breaching species barriers and eroding traditional control boundaries (2). The aspiration for “zero infection” has yielded to long-term societal anxiety about “living with risk.” Finally, from the Melamine-tainted Infant Formula and the Soil-pit Pickled Vegetables to the recent Lead-poisoning in a Gansu Kindergarten (3), the frequent food safety incidents have continuously sounded the alarm, spreading the fear of “risk in the mouth” to every meal. The convergence of these triple crises amplifies public uncertainty about the future and fundamentally reshapes consumption priorities: safety, health, and sustainability have become overriding imperatives. Consequently, safety, organic, and traceable foods have transitioned from optional choices to necessities.

Amid the convergence of multiple crises, individuals’ sensitivity is rooted in the fear of death (4). According to TMT, the awareness of mortality triggers anxiety, which individuals manage by developing specific defense mechanisms. These mechanisms serve to provide a sense of personal value and symbolic immortality (5, 6). Prior studies have posited that two key psychological buffers against mortality salience are self-esteem and cultural worldview (5, 7, 8). Self-esteem emphasizes the promotion and protection of self-worth, driving individuals to seek self-affirmation that “I deserve to live.” This manifests as a heightened preference for safer and healthier products. And cultural worldview embeds individual actions within a larger narrative of “protecting the collective and future generations,” thereby affording a sense of symbolic immortality. It is expressed through choices favoring goods that are environmentally sound, altruistic, and sustainable. Together, these defense mechanisms help explain why individuals gravitate toward green food consumption in times of crisis.

To our knowledge, previous literature on the motivations for green food consumption has predominantly focused on product attributes (9), perceived value (10), and public opinion (11), while rarely considering negative emotions such as death anxiety and fear. Consequently, the psychological motives of self-protection and meaning-making under threat remain underexplored. As for “green food,” which integrates the dual attributes of personal health and safety (functional value) and environmental sustainability (symbolic value) (10). According to Tang et al. (12), green food is a certified food category produced, processed, stored, and transported in line with the principles of sustainable development, characterized by core attributes of health and safety, low pollution, and ecological friendliness. This definition is consistent with the cognitive scope of green food among Chinese consumers. In crisis contexts, avoidance motivation for self-protection aligns with the functional attributes of green foods, whereas approach motivation for meaning-making aligns with their symbolic attributes. Therefore, a perspective of approach and avoidance motivation offers a valuable framework for revealing how mortality salience governs green food consumption.

In addition, under stress or death-related threats, individuals rely on “mental shortcuts.” The Stereotype Content Model (SCM) posits that warmth and competence are the two most fundamental and rapid dimensions people use to evaluate products and brands (13). When green foods signal benevolence, ethics, and care, they elicit altruistic approach motivation; when they emphasize technology, efficiency, and safety, they trigger self-protective avoidance motivation.

Taken together, this paper pursues three primary objectives: (1) to explore the effect of mortality salience on consumers’ intention to purchase green food; (2) to investigate the dual-path mechanism (approach vs. avoidance) through which mortality salience drives green food purchase intention; (3) to test the moderating roles of perceived warmth and competence in this dual-path effect. Amidst growing contemporary health challenges, this paper offers a fresh perspective on how mortality salience influences the intention to purchase green food. More importantly, the research not only promotes the level of social well-being but also accelerates the transition to sustainable consumption. In addition, this study equips firms with targeted strategies for developing green foods that integrate safety with symbolic value.

## 2 Literature review and hypothesis development

**FIGURE 1:**
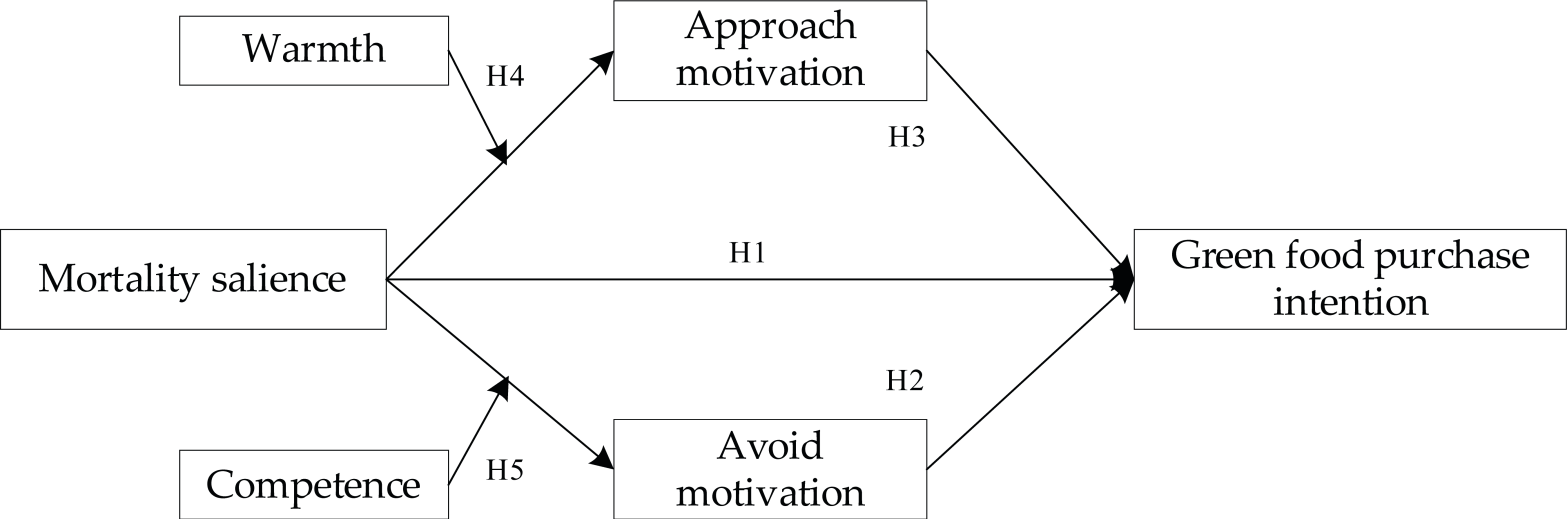
The conceptual framework.

To dissect the underlying psychological mechanisms through which mortality salience shapes consumers’ green food purchase intention, we integrate Terror Management Theory (TMT), Approach-Avoidance Motivation Theory (AATM), and the Stereotype Content Model (SCM) to propose a dual-path moderated mediation model (Figure 1). Specifically, we hypothesize that mortality salience exerts a positive direct effect on green food purchase intention (H1), which is mediated by two parallel pathways: avoidance motivation (H2) and approach motivation (H3). Furthermore, perceived warmth moderates the approach motivation pathway (H4), while perceived competence moderates the avoidance motivation pathway (H5). The following sections elaborate the theoretical rationale for each hypothesis, with continuous reference to the corresponding paths in Figure 1.

### 2.1 Mortality salience and green food purchase intention

H1. Mortality salience positively affects green food purchase intention

Frequent pandemics, climate change, and human-induced safety incidents are existential threats that humanity must simultaneously grapple with, and they also serve as mortality salience triggers (6, 14). According to Terror Management Theory (TMT), the unresolvable conflict between humans’ innate desire to live and consciousness of death has the potential to lead to existential anxiety, fear of death, and thanatophobia (6, 8, 15). Especially when the real death event is stimulated, the fear and anxiety of people under mortality salience are overwhelming (4).

Research on Terror Management Theory (TMT) has found that individuals under mortality salience strive to develop an “anxiety buffering system” to suppress or escape the terror (4, 14). Consumption behavior is a critical and tangible external manifestation of this anxiety-buffering process, as consumer tend to select products that align with their psychological needs for safety, security, and meaning-making (16). Green food, as a product category associated with higher safety profiles, lower health risks, and environmental sustainability (17–19), is inherently suited to alleviate the existential anxiety triggered by mortality salience. It is important to clarify that unlike ordinary safety foods, green foods are subject to stringent mandatory third-party certification and a full-process traceable quality control system. They not only provide consumers with more robust safety guarantees but also align with consumers’ long-term aspirations for environmental protection and ecological sustainability.

### 2.2 Mediating role of approach-avoidance motivation

H2. Avoidance motivation serves as an intermediary mechanism linking mortality salience to green food purchase intention.

H3. Approach motivation serves as an intermediary mechanism linking mortality salience to green food purchase intention.

According to the Approach-avoidance Motivation Theory (AATM), seeking benefits and avoiding harm constitutes the most fundamental and critical response of all organisms to environmental stimuli (19). When encountering external stimuli, individuals typically develop two motivational tendencies: seeking benefits (approach motivation) and avoiding harm (avoidance motivation). These distinct motivational orientations determine variations in behavioral responses (20, 21). Theoretically, approach and avoidance motivations can co-activate within the same individual and behavioral context (21).

From the perspective of individual motivational response to external threats, the approach-avoidance motivation triggered by survival-related stimuli is inherently aligned with the operational logic of the two-core anxiety-buffering defense mechanisms (self-esteem and cultural worldview) under mortality salience proposed by Terror Management Theory (TMT). Especially in the crisis context of food safety incidents, pandemics, and climate disasters, they present a relationship as follows.

Specifically, the self-esteem defense, the core proximal anxiety-buffering system in TMT, is activated to address immediate survival threats when mortality is made salient (5, 7). As Ma et al. (4) noted, self-esteem emphasizes self-worth. Its core functional goal is to maintain the individual’s sense of self-worth through protecting the physical safety and health of themselves and their families (22–24). This goal is fully consistent with the core connotation of avoidance motivation: evading potential risks, minimizing harm, and safeguarding existing well-being (2, 25). Empirically, Landau and Greenberg (26) confirmed that in high-risk, survival-relevant contexts, self-esteem defense is consistently associated with risk-avoidant, the immediate priority shifts to self-preservation.

In the context of green food consumption, the core goal of avoidance motivation is to avoid potential health risks to oneself and one’s family members. First, public fear of viruses or perceived scarcity of supplies compels heightened concern for oneself or closely affiliated individuals (27). Second, under mortality threat, consumers exhibit an increased likelihood of rejecting products that pose potential health risks to themselves or their family members (28). Against the backdrop of frequent environmental crises and food safety incidents, consumer trust in ordinary foods has been further eroded (29), and the “default safe” status established solely based on basic regulatory compliance is no longer convincing. By contrast, green foods can provide more robust safety guarantees, thus becoming the preferred “safe haven” for risk-averse consumers (30, 31). Taken together, heightened death awareness intensifies self-preservation motivation. Green food consumption may primarily function as an expression of this self-protective impulse.

Furthermore, the cultural worldview defense, the core distal symbolic anxiety-buffering system in TMT, is activated to alleviate long-term existential anxiety by granting individuals a sense of symbolic immortality (6). Its core functional goal is to maintain shared cultural norms through prosocial and pro-environmental behaviors that promote collective and long-term ecological well-being, aligning with approach motivation: pursuing positive outcomes, advancing collective welfare, and realizing meaningful value (32–35). Empirically, this mapping is validated by prior TMT research in pro-environmental and prosocial contexts. Grant and Wade-Benzoni (25) found that mortality salience activates intergenerational altruism and prosocial approach motivation, an effect fully mediated by adherence to cultural worldviews that prioritize collective and environmental welfare.

In the context of green consumption, approach motivation refers to a rewards-seeking motivational tendency centered on meaning-making, whose core goal is to pursue positive outcomes for society, the environment and future generations, and to realize transcendent symbolic value. Under mortality threat, mortality threat activates desires for lasting significance. Individuals buffer death anxiety by extending their contributions into the future while assuming personal responsibility for promoting the well-being of others and future generations (36, 37). This aligns with the orientation of green consumption toward anticipated environmental welfare (38). As for green food consumption, this means that mortality salience will activate the cultural worldview defense, and then generate approach motivation to promote environmental sustainability and social welfare through purchasing green food with ecological attributes.

Notably, the above dominant matching relationship does not mean that the two mechanisms are completely independent or isolated. In this study, we focus on the dominant motivational tendency of the two defense mechanisms in the specific green food consumption context under mortality salience, while fully acknowledging the potential overlap and multi-faceted manifestations of the two psychological pathways.

### 2.3 Moderating role of perceived warmth and competence

H4. Perceived warmth moderates the effect of mortality salience on approach motivation.

H5. Perceived competence moderates the effect of mortality salience on avoidance motivation.

The Stereotype Content Model (SCM) posits that individuals rely on “mental shortcuts” in information-scarce or ambiguous decision-making. It distills cues into two core dimensions: warmth (friendliness, trustworthiness) and competence (intelligence, efficiency) (13, 39, 40). These highly predictive dimensions are widely used in marketing to examine consumer perceptions of brands and firms (41–43).

This study applies SCM to green consumption for two key reasons:

First, limited and ambiguous green market information, coupled with consumers’ lack of expertise to independently evaluate green products, increases their reliance on SCM as a cognitive heuristic (40, 44). Second, green consumption addresses both functional utility needs and environmental ethical concerns. SCM’s dual dimensions, self-interest-driven competence and morality-based warmth, effectively explain the green consumption attitude-behavior gap (45).

Consumer behavior research grounded in the Stereotype Content Model (SCM) demonstrates that the two dimensions of warmth and competence map onto asymmetric, non-interchangeable motivational orientations, which originate from adaptive needs during human evolution. Specifically, perceived warmth primarily activates approach motivation (19). Warmth signals prompting individuals to actively approach and affiliate with warm targets (39). In the domain of consumer behavior, warmth embodies consumers’ moral appeals (40, 46). When food companies demonstrate harmonious, benevolent, and altruistic intentions through environmental responsibility, consumers experience heightened perceived warmth (47, 48). This ethical stance aligns with consumers’ cultural worldview, which emphasizes environmental protection and collective well-being, and consequently amplifies their motivation to safeguard the environment and enhance social welfare.

Correspondingly, perceived competence primarily triggers avoidance motivation (19). Under threatening or fearful states, consumers tend to narrow their focus to immediate information and exhibit heightened self-concern (49). When food companies demonstrate superior competence in areas such as food safety, ecological conservation, and green technology, particularly by meeting and exceeding industry standards, consumers perceive the green brand as more competent (2, 19). This competence perception enhances consumers’ perceived efficacy in coping with existential threats, subsequently activating risk-averse behavioral tendencies centered on self-protection in uncertain environments. The competence dimension of green food brands fulfills consumers’ critical defensive needs by addressing their functional requirements (10).

In addition, under threatening states, the motivational differences elicited by warmth and competence are further amplified (2). At this point, cognitive resources narrow, eliminating ambiguous motivational responses. Alternative matching patterns are theoretically and empirically implausible in this context: high competence without warmth is perceived as threatening and exploitative, which would not elicit approach behavior; high warmth without competence is seen as well-meaning but ineffective, which cannot satisfy the urgent risk-reduction demands triggered by mortality salience.

## 3 Materials and methods

### 3.1 Study design

As previously discussed, mortality salience triggers two defensive mechanisms: self-esteem and cultural worldview (6). These mechanisms elicit dual motivational responses: approach motivation manifests as heightened expectations of future environmental quality and collective well-being, whereas avoidance motivation reflects a desire to maintain health and evade risk (2, 19). These dual motivations jointly increase individuals’ preference for green food. Furthermore, this paper examines the roles of perceived warmth and competence within these pathways.

Therefore, we conducted four experiments to test the hypotheses proposed in the previous stage. Study 1 used a two-way analysis of variance (ANOVA) to confirm the effect of mortality significance on green food purchase intention (H1). Study 2 employed a bootstrap mediation analysis to assess the mediating roles of approach and avoidance motivation (H2, H3). Studies 3 and 4 applied moderated mediation analyses to investigate the moderating effects of perceived warmth and competence (H4, H5). Table 1 provides a structured summary of the complete research design for all four experiments.

**TABLE 1:** Summary of research design and hypothesis allocation.

Study ID	Hypotheses tested	Experimental design	Sample size
Study 1	H1	Single-factor between-subjects design (high mortality salience vs. low mortality salience)	131
Study 2	H1, H2, H3	Single-factor between-subjects design (high mortality salience vs. low mortality salience)	135
Study 3	H4	2 (mortality salience: high/low) × 2 (perceived warmth: high/low) between-subjects design	142
Study 4	H5	2 (mortality salience: high/low) × 2 (perceived competence: high/low) between-subjects design	146

### 3.2 Experimental manipulation

#### 3.2.1 Mortality salience manipulation

Previous studies have established that narrative recall and video clips can capture the emotion of different features (2, 50). We manipulated mortality salience by having the subjects watch edited videos. According to (4, 5, 51), participants were randomly assigned to two experimental conditions manipulating mortality salience (high vs. low) and viewed corresponding video stimuli. Those in the high mortality salience condition were exposed to a video featuring natural disasters (e.g., typhoons, floods), child fatalities resulting from food safety incidents, and grim news reports amidst the pandemic. A brief documentary segment depicting routine dental treatment was presented to the low mortality salience group. Following the video stimuli, participants completed a 6-item mortality salience scale, adapted from (4, 52).

#### 3.2.2 Perceived warmth manipulation

**FIGURE 2:**
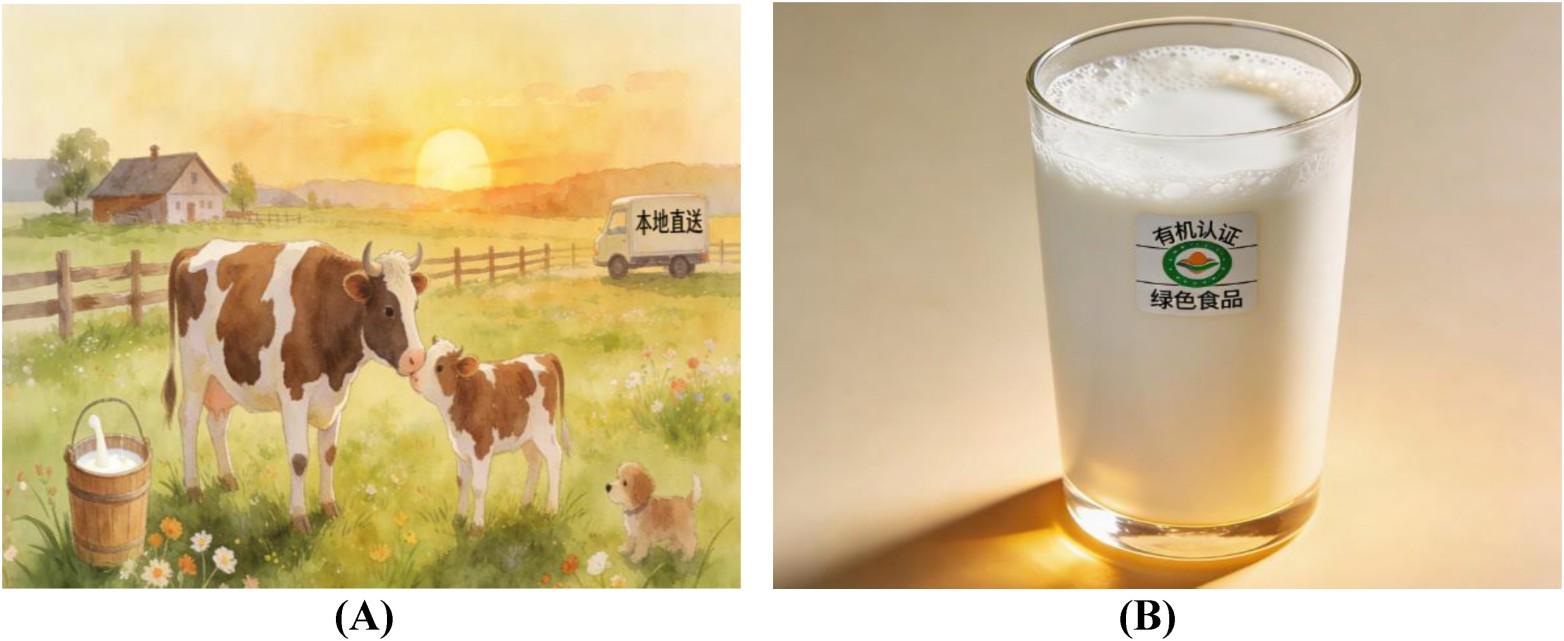
Experimental stimuli for green food warmth perception. **(A)** High warmth condition. **(B)** Low warmth condition.

For perceived warmth manipulations, we developed text-based scenarios adapted from Kolbl et al. (41) and tailored to the green food context. The scenarios featured the organic yogurt as the experimental product. In the high warmth perception scenario of green food, the advertisement of organic yogurt emphasized affective and communal appeals with the taglines: “Every spoonful is filled with love,” “Our organic yogurt is lovingly crafted on local farms, with every drop carrying the care we give our own family,” and “We believe true health arises from warm connections among people.” The packaging design highlighted “handmade,” “small-batch,” and “harmony between humans and nature,” and displayed an image of two cows, the calf cuddling up to its mother and the puppy running around happily in a warm family-oriented farm environment (see Figure 2A). In the low warmth perception scenario of green food, the advertisement of organic yogurt emphasized functional and regulatory appeals with the taglines: “Every cup meets strict quality standards,” “Our organic yogurt is strictly produced in certified facilities, with every batch passing rigorous third-party quality inspections,” and “We believe true safety comes from strict compliance with rules.” The packaging design highlighted “organic certified,” “green food,” and “compliance with national standards,” and displayed an image of a yogurt cup, the official certification logos printed clearly and no extra decorative elements added in a plain minimalist product presentation (see Figure 2B).

#### 3.2.3 Perceived competence manipulation

**FIGURE 3:**
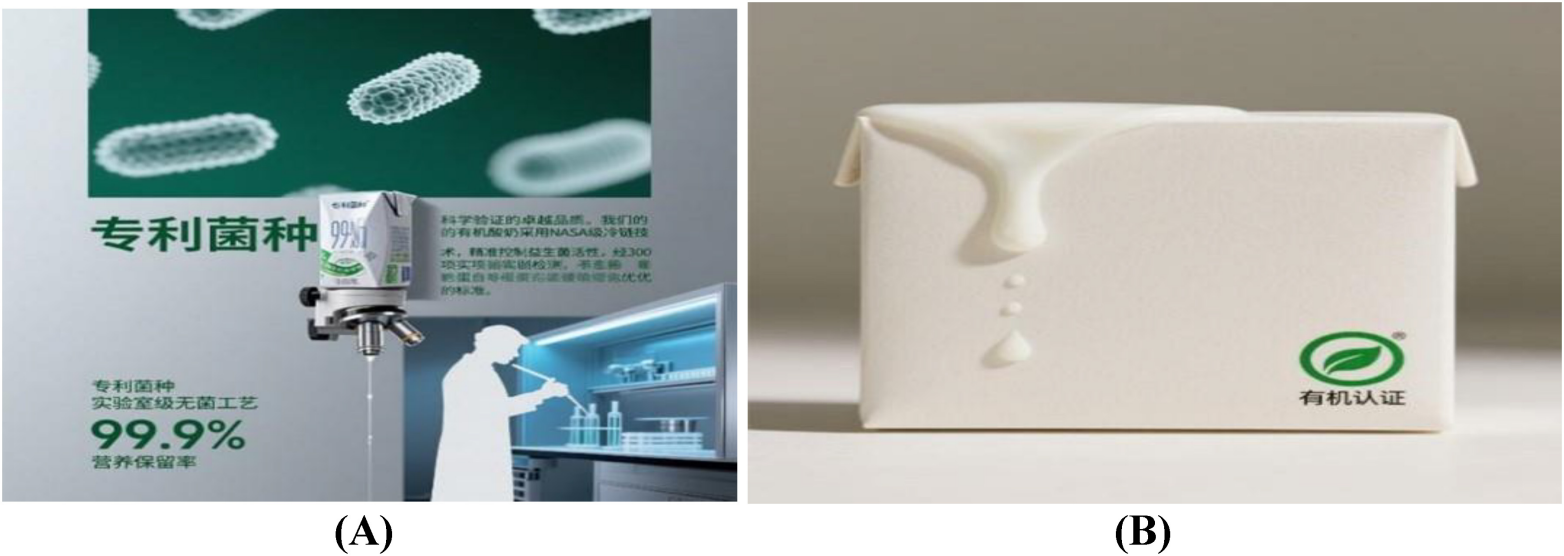
Experimental stimuli for green food competence perception. **(A)** High competence condition. **(B)** Low competence condition.

For perceived competence manipulation, we likewise constructed the scenarios that were adapted from Kolbl et al. (41) and featured the organic yogurt as the experimental product. In the high competence scenario of green food, the advertisement of organic yogurt stressed scientific rigor and superior performance through the taglines: “Scientifically validated excellence,” and “Our organic yogurt employs NASA-grade cold-chain technology to precisely control probiotic activity and under-goes 300 laboratory tests to ensure every gram of protein meets optimal standards.” The packaging showcased “patented bacterial strains,” “laboratory-grade aseptic processing,” and “99.9% nutrient retention,” accompanied by an image of probiotics under a microscope and a scientist working in a laboratory (see Figure 3A). In the low competence scenario of green food, the advertisement of organic yogurt stressed basic compliance and minimum requirements through the taglines: “Basic industry standards met,” and “Our organic yogurt uses standard commercial cold-chain technology to generally maintain probiotic activity and undergoes 3 routine factory tests to ensure every gram of protein meets minimum standards.” The packaging showcased “common bacterial strains,” “regular factory processing,” and “80% nutrient retention,” accompanied by an image of a yogurt box with organic certification and no extra decorative elements (see Figure 3B).

### 3.3 Measurement of variables

All variables were measured using mature and validated scales, and all items were scored on a 7-point Likert scale (1 = strongly disagree, 7 = strongly agree).

Mortality salience was measured with a 6-item scale adapted from Ma et al. (4) and Liu et al. (52). Representative items included “The content just now reminded me of death,” “The content just now made me feel the threat to my survival,” “The content just now made me feel the threat to my health,” and “I worry that I might die as a result of an epidemic,” and so forth.

Both perceived warmth and perceived competence were measured using 3-item scale adapted from Fiske (39) and Aaker et al. (46). For perceived warmth, sample items were: “It makes me feel friendly,” “It evokes associations with my family’s health and happiness,” and “It conveys the tender feeling of harmony between humans and nature.” For perceived competence, sample items were: “It makes me feel the product is nutritious and healthy,” “It reflects advanced green technology,” and “It indicates greater food safety.”

Approach motivation was measured using a 5-item prosocial and pro-environmental scale originally developed by Grant and Wade-Benzoni (25). Representative items include “I care about the healthy development of others/society/the environment,” “I want to exert effort to positively influence others/society/the environment,” and “I believe benefiting others/society/the environment is both important and meaningful.” Avoidance motivation was measured with the 5-item scale developed by Su et al. (2) and Sun et al. (53). Sample items are “I actively avoid situations that may damage my health or my family,” “When faced with threats, I strive to prevent health damage to me or my family,” “I would never risk trying any potentially problematic things,” and “I avoid engaging in any activities that may involve risks,” and so forth.

Green food purchase intention was measured using the 5-item scale developed by Wang et al. (54). Sample items include “I intend to purchase green food,” “I would recommend others to buy green food,” “I will buy green food in the future,” and so forth.

### 3.4 Participants and procedure

#### 3.4.1 Participants

All experiments were conducted on the sports field of a public university in Wuhan during weekends. As the field was open to the public, we were able to recruit a diverse sample, including university students, nearby residents, and other community members, thereby enhancing the external validity of the study. However, it should be noted that all participants were recruited from Wuhan, Hubei Province, China, and the sample is geographically constrained, which limits the generalizability of the research findings. As a token of appreciation, each participant received a plush toy upon completing an experimental session. A total of 990 person-times were recorded across the four studies. After excluding 77 invalid entries from individuals who participated in more than one experiment, 913 unique valid samples were retained for subsequent analysis.

Before the formal experiment, we confirmed that all participants were physically and psychologically healthy, and we also provided a clear explanation of the concept of green food to ensure participants had a consistent and accurate understanding of the core research object. The studies involving human participants were reviewed and certified by the relevant research administration department of our university. Written informed consent with a clear data privacy clause was obtained from each participant, and all experimental procedures were carried out in strict compliance with the ethical standards of the Declaration of Helsinki.

#### 3.4.2 Procedure

On the basis of the preliminary manipulation checks for mortality salience, perceived warmth and perceived competence, this study formally conducted four experiments to test the proposed hypotheses.

In study 1, we firstly conducted *a priori* power analysis (to determine the minimum required sample size) using G*Power 3.1 (55). According to the calculations, a sample of at least 128 individuals is required [moderate-size effect (α = 0.05), and power (1−β = 0.8)]. Considering the unstable factors in the experiments, a total of 131 experimental participants (72 males and 59 females) were randomly divided into two groups. Following the video, participants completed scales measuring green food purchase intention.

In study 2, we tested the mediating roles of approach and avoidance motivations. Consistent with Study 1, a total of 135 participants (77 males and 58 females) were randomly divided into two groups. After the video, participants were asked to finish scales of green food purchase intention, approach motivation, and avoidance motivation.

Studies 3 and 4 employed a 2 × 2 between-subjects design to examine the moderating effects of perceived warmth and competence, respectively. For the PROCESS Model 7 moderated mediation analysis, the *a priori* analysis indicated a minimum total sample size of 103 to detect a moderate f^2^ = 0.15 indirect effect with 0.8 power. Our final sample sizes (*N* = 142 for Study 3; *N* = 146 for Study 4) both meet and exceed this requirement, ensuring adequate statistical power for testing the complex conditional indirect effects in the dual-path model. After viewing the video and reading the corresponding stimulus materials, participants completed the relevant items.

In addition, previous studies suggest that the effect of MS manipulation on the dependent variable becomes more accessible and effective when there is a delay between the manipulation and the measurement of the dependent variable (35). We followed the approach of Dunn et al. (56) and Jeong et al. (57), by having all participants complete a brief cognitive distraction task (writing down the current date, day of the week, time, and postal code) after the mortality salience manipulation, allowing for more distal responses to death awareness.

### 3.5 Manipulation check

For mortality salience, 130 participants were recruited for the manipulation check. After viewing the video stimuli, participants completed the mortality salience scale. Cronbach’s α for this scale was 0.925. An independent-samples *t*-test confirmed that participants in the high mortality salience condition reported significantly greater mortality salience (*M* = 5.34, *SD* = 1.21) than those in the low-salience condition (*M* = 4.25, *SD* = 0.87), *t* (128) = 5.88, *p* < 0.001. These results verify the effectiveness of the experimental manipulation, supporting its use in subsequent analyses.

For perceived warmth, 116 individuals participated in the manipulation check. After reading the text materials, participants finished the perceived warmth scale. Cronbach’s α for this scale was 0.80. The result showed that the high-warmth group reported significantly greater warmth toward the green food compared to the low-warmth group [*M*-high = 5.30, *SD* = 0.87 vs. *M*-low = 3.60, *SD* = 0.77; *t* (114) = 11.13, *p* < 0.001], indicating successful manipulation of perceived warmth.

For perceived competence, the manipulation check was also conducted with 113 participants. Cronbach’s α for this scale was 0.90. Participants in the high competence group perceived significantly greater competence of the green food than those in the low-competence group [*M*-high = 5.18, *SD* = 0.54 vs. *M*-low = 3.77, *SD* = 0.56; *t* (111) = 13.51, *p* < 0.001], verifying the effectiveness of perceived competence manipulation.

## 4 Results

### 4.1 Study 1

First, we assessed scale reliability. The green food purchase intention scale yielded a Cronbach’s α of 0.80, indicating excellent internal consistency. Subsequently, ANOVA was conducted to examine the direct effect of mortality salience on green food purchase intention. Participants in the high mortality salience condition (*M* = 5.22, *SD* = 0.85) showed significantly greater intention to purchase green foods than those in the low salience condition (*M* = 4.00, *SD* = 0.72), *F* (1,129) = 78.54, *p* < 0.001, η^2^ = 0.38. After controlling for participants’ gender (*F* = 4.05, *p* = 0.05), age (*F* = 0.71, *p* = 0.40) and education background (*F* = 4.22, *p* = 0.04), the main effect remained significant (*p* < 0.001). The *post hoc* sensitivity analysis showed that the actual sample size (*N* = 131) could reliably detect a minimum effect size of Cohen’s *d* = 1.55 (two-tailed). Meaning the sample is sufficiently powered to detect even large main effects. Thus, Hypothesis 1 was supported.

According to the results of study 1, individuals are significantly more inclined to purchase green foods under high mortality salience, a pattern consistent with both self-protective (self-esteem) and social value realization (cultural worldview) mechanisms. Specifically, reminders of mortality (e.g., climate change, natural disasters) increase health concerns. The perception that green food addresses these concerns enhances its appeal. At the same time, mortality salience intensifies individuals’ desire to contribute to the sustainable development of nature and society. The inherent sustainability of green consumption aligns with this aspiration, further motivating green food purchases.

### 4.2 Study 2

Prior to formal hypothesis testing, we conducted a complete reliability and validity test for core variables involved in this study: approach motivation, avoidance motivation, and green food purchase intention, with no missing data for all participants (*N* = 135). First, the reliability test showed that the Cronbach’s α was 0.88 for approach motivation, 0.88 for avoidance motivation and 0.92 for green food consumption, all above the critical value of 0.7, indicating excellent internal consistency. Second, confirmatory factor analysis (CFA) showed that the hypothesized three-factor model had a good fit with the data: χ^2^/df = 1.82, CFI = 0.96, TLI = 0.95, RMSEA = 0.07, SRMR = 0.07. The composite reliability (CR) of all variables ranged from 0.89 to 0.93 (approach motivation = 0.89, avoidance motivation = 0.92, green food consumption = 0.93). The average variance extracted (AVE) ranged from 0.63 to 0.70 (approach motivation = 0.63, avoidance motivation = 0.69, green food consumption = 0.70). As shown in Table 2, the HTMT values between all pairs of latent variables ranged from 0.56 to 0.82, all below the conservative threshold of 0.85, further confirming that the core constructs had satisfactory discriminant validity and were empirically distinct from each other. Finally, we tested for common method bias using Full Collinearity Approach for verification. The results show that the Variance Inflation Factor (VIF) values of all latent variables range from 1.58 to 1.72, which are all far below the strict threshold of 3.3, indicating no serious common method bias in this study.

**TABLE 2:** HTMT results for discriminant validity of core latent variables.

Latent variable	Approach motivation	Avoidance motivation	Green food purchase intention
Approach motivation	–	0.56	0.65
Avoidance motivation	0.56	–	0.82
Green food purchase intention	0.66	0.82	–

Analysis of variance indicated a significantly higher intention to purchase green foods among participants in the high mortality salience group (*M* = 5.07, *SD* = 0.75) relative to those in the low salience condition (*M* = 2.68, *SD* = 0.72), *F* (1,133) = 357.49, *p* < 0.001, η^2^ = 0.73. These results corroborate Hypothesis 1.

**FIGURE 4:**
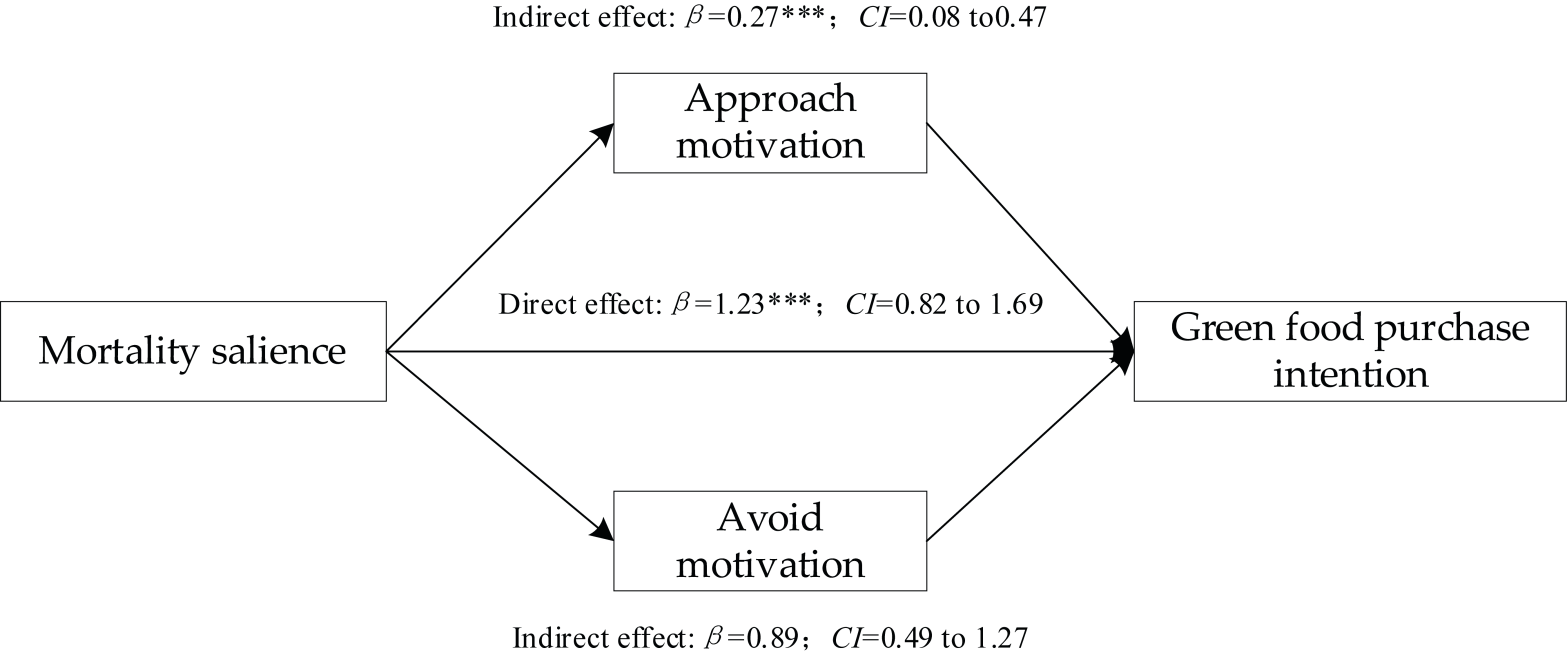
Mediating effect of approach motivation and avoidance motivation. ***Indicates a statistically significant difference at the *p* < 0.001 level in the respective comparisons presented.

Subsequently, we tested the mediating roles of approach and avoidance motivations:

(1) ANOVA indicated a significant increase in approach motivation among participants under high mortality salience (*M* = 4.78, *SD* = 1.01) relative to the low salience condition (*M* = 2.98, *SD* = 0.95), *F* (1,133) = 114.53, *p* < 0.001, η^2^ = 0.46. Likewise, in the high mortality group (*M* = 5.02, *SD* = 0.77), the score for the avoidance motivation was higher than that in the low mortality group (*M* = 2.70, *SD* = 0.73), *F* (1,133) = 322.69, *p* < 0.001, η^2^ = 0.71]. These findings indicate that mortality salience simultaneously heightens both approach and avoidance motives.

(2) Regression analysis showed that approach and avoidance motivation exerted a significant positive effect on green food purchase intention (β = 0.29, *t* = 5.09, *p* < 0.001; β = 0.68, *t* = 12.44, *p* < 0.001).

(3) According to Hayes (58), we adopted PROCESS Model 4 with 5,000 bootstrap samples to test the mediating roles of approach and avoidance motivations in the pathway from mortality salience to green food purchase intention. As shown in Figure 4 and Table 3, both approach motivation (β = 0.27, 95% CI [0.08, 0.47]) and avoidance motivation (β = 0.89, 95% CI [0.49, 1.27]) exerted significant indirect effects, providing full support for H2 and H3. After controlling for the dual mediators, the direct effect of mortality salience on purchase intention (β = 1.23, 95% CI [0.82, 1.69]) was significant, indicating that the two motives play a mediation effect between mortality salience and green food purchase intention.

**TABLE 3:** Mediating analysis of approach motivation and avoidance motivation.

Types of effects	Mediating variable	Effect value	*SE*	*P*	95% CI	
					LLCI	ULCI
Direct effect	–	1.23^***^	0.19	<0.001	0.82	1.69
Mediating effect	Approach motivation	0.27^***^	0.10	<0.001	0.08	0.47
	Avoidance motivation	0.89^***^	0.17	<0.001	0.49	1.27

^***^Means *P*-value less than 0.001.

Taken together, the dual-pathway model shows that both approach and avoidance motivations mediate the effect of mortality salience on green food consumption. On the one hand, acute mortality threats such as climate disasters heighten self and family protective motives, leading individuals to purchase organic, healthy, and safe green foods to safeguard physical well-being. On the other hand, the same threats activate broader social and environmental protection goals, motivating consumers to buy and encourage others to buy green foods to reduce pollution and environmental degradation.

### 4.3 Study 3

First of all, we assessed the reliability of scales. Cronbach’s α was 0.86 for approach motivation and 0.72 for green food consumption, indicating good internal consistency. The convergent validity test showed that the CR of approach motivation is 0.91, and the CR of green food consumption is 0.79. The AVE of approach motivation is 0.70, and the AVE of green food consumption is 0.52, all meeting the standard requirements.

**FIGURE 5:**
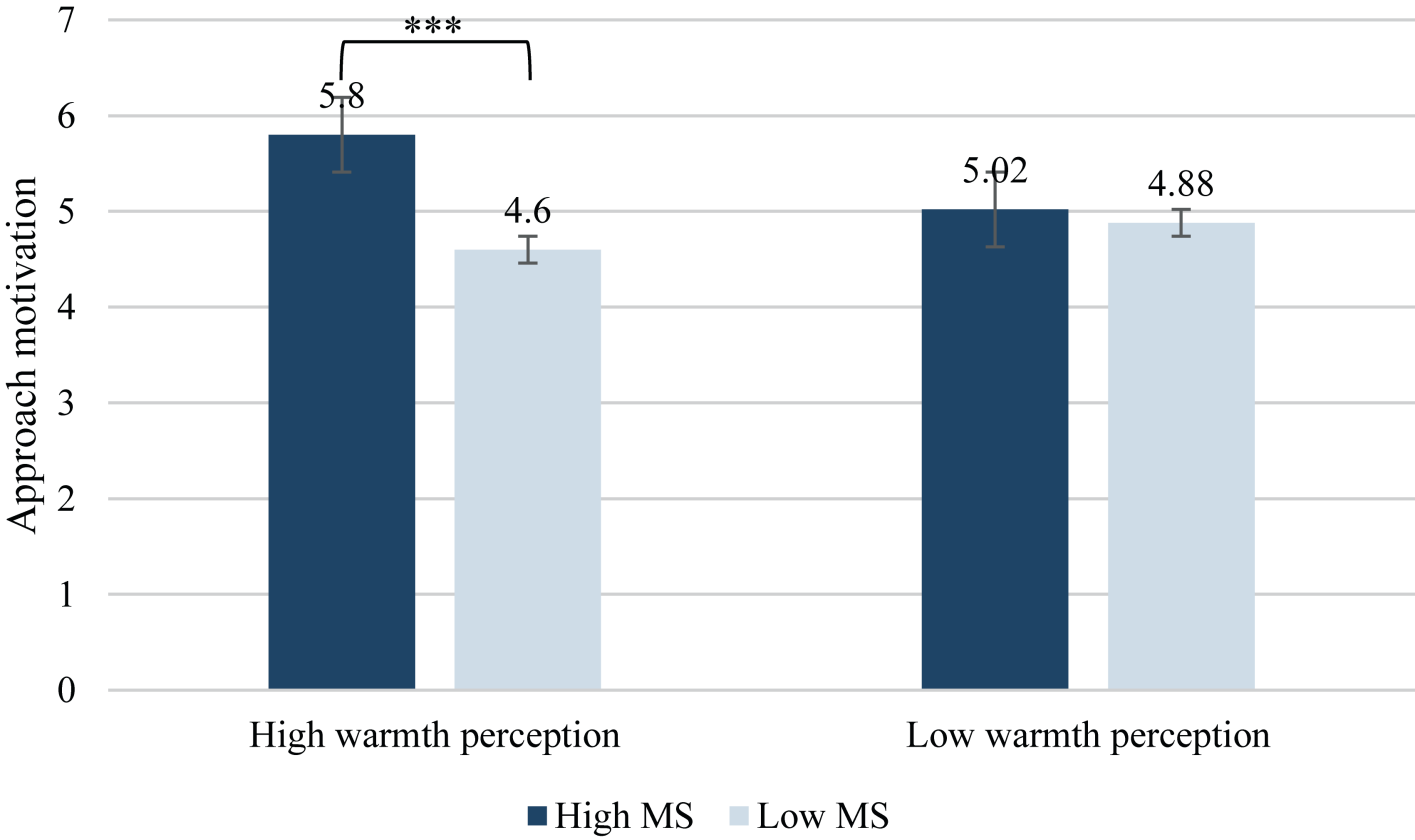
Moderating effect of perceived warmth. ***Indicates a statistically significant difference at the *p* < 0.001 level in the respective comparisons presented.

The inter-group experiment of indicated that when the green food was described as high in warmth, participants in the high mortality group (*M* = 5.80, *SD* = 0.20) reported significantly stronger approach motivation compared to those in the low mortality group (*M* = 4.60, *SD* = 0.82), *F* (1,65) = 69.92, *p* < 0.001, η^2^ = 0.53. In contrast, when warmth cues were absent, the difference between the two mortality salience groups was not significant (*M*-high = 5.02, *SD* = 0.66; *M*-low = 4.88, *SD* = 0.64; *F* (1,73) = 0.85, *p* = 0.36, η^2^ = 0.11. The results are illustrated in Figure 5.

Furthermore, we conducted a moderated mediation analysis using PROCESS Model 7 (58). Green food consumption served as the dependent variable, mortality salience as the independent variable (high = 1, low = 0), approach motivation as the mediator, and warmth as the moderator (high = 1, low = 0).

When the green food was perceived as high in warmth, the mediating effect of the approach motivation was significant (effect = 0.54, 95% CI [0.33, 0.75]). In contrast, under low warmth conditions, the mediating effect was non-significant (95% CI [−0.07, 0.21]). The overall index of moderated mediation was 0.48 (95% CI [0.25, 0.71]) (see Table 4). Hypothesis H4 is therefore supported: only when green food is framed as high in warmth does mortality salience heighten approach motivation, which in turn increases consumers’ green food purchase intention.

**TABLE 4:** The moderated mediation role of approach motivation.

Types of effects	Mediating variable	Moderating variable	Effect value	*SE*	*P*	95% CI	
						LLCI	ULCI
Direct effect	–	–	0.10	0.08	0.21	−0.06	0.26
Mediating effect	Approach motivation	High warmth	0.54^***^	0.11	<0.001	0.33	0.75
		Low warmth	0.06	0.07	0.34	−0.07	0.21

^***^Means *P*-value less than 0.001.

In addition, the *post hoc* sensitivity analysis showed that the actual sample size (*N* = 142) could reliably detect a minimum effect size of Cohen’s *d* = 0.44 (two-tailed). Meaning the sample is sufficiently powered to detect even detect even sub-moderate effect.

### 4.4 Study 4

Following the same procedure as in Study 3, we examined the moderating role of competence. Firstly, we assessed the reliability of the avoidance motivation scale (Cronbach’s α = 0.92) and the reliability of the green food consumption scale (Cronbach’s α = 0.81), indicating good internal consistency. The convergent validity test showed that the CR of variables ranged from 0.79 to 0.83, and the AVE ranged from 0.53 to 0.58, all meeting the standard requirements.

**FIGURE 6:**
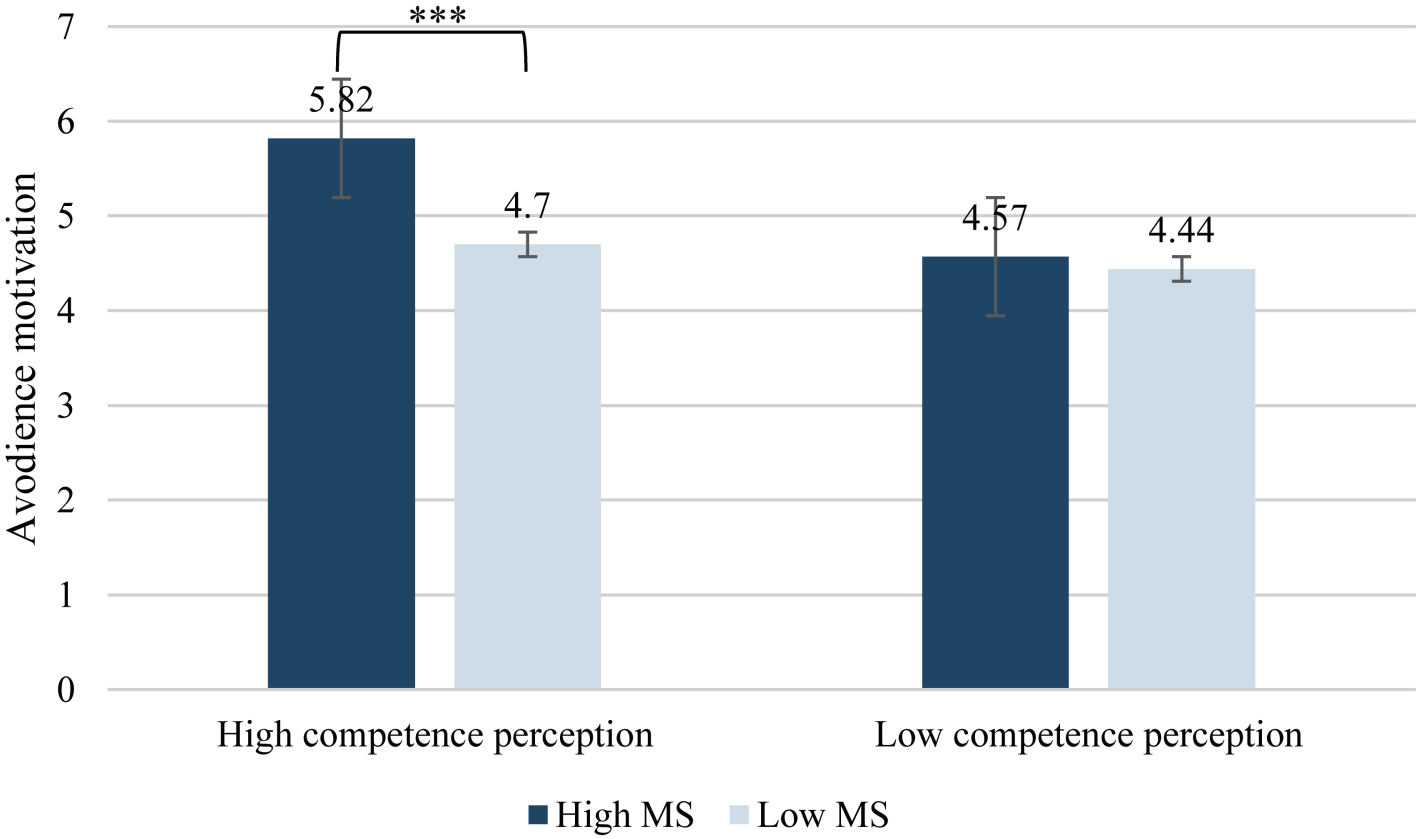
Moderating effect of perceived competence. ***Indicates a statistically significant difference at the *p* < 0.001 level in the respective comparisons presented.

Then the inter-group experiment showed that when the green food was portrayed as high in competence, participants under high mortality salience (*M* = 5.82, *SD* = 0.62) reported significantly stronger avoidance motivation compared to those in the low mortality group (*M* = 4.70, *SD* = 0.59), *F* (1,70) = 63.20, *p* < 0.001, η^2^ = 0.47. When competence cues were absent, the difference between the two mortality salience groups was nonsignificant [*M*-high = 4.57, *SD* = 0.74; *M*-low = 4.44, *SD* = 0.68; *F* (1,74) = 0.53, *p* = 0.47, η^2^ = 0.01]. The results are illustrated in Figure 6.

Consistent with Study 3, we again employed PROCESS Model 7 (58) to test the moderated mediation. In the regression model, green food purchase intention served as the dependent variable, mortality salience as the independent variable (high = 1, low = 0), avoidance motivation as the mediator, and perceived competence as the moderator (high = 1, low = 0). When the green food was perceived as high in competence, the indirect effect of mortality salience on purchase intention through avoidance motivation was significant (effect = 0.38, 95% CI [0.19, 0.48]). In contrast, when competence was low, this indirect effect was nonsignificant (95% CI [−0.05, 0.14]). The index of moderated mediation for avoidance motivation was 0.27 (95% CI [0.14, 0.41]) (see Table 5). Hypothesis 5 is therefore supported: when green food is positioned as highly competent, mortality salience heightens consumers’ avoidance motivation, which in turn increases their purchase intention.

**TABLE 5:** The moderated mediation role of avoidance motivation.

Types of effects	Mediating variable	Moderating variable	Effect value	*SE*	*P*	95% CI	
						LLCI	ULCI
Direct effect	–	–	0.11	0.07	0.11	−0.02	0.25
Mediating effect	Avoidance motivation	High competence	0.38^***^	0.08	<0.001	0.19	0.48
		Low competence	0.04	0.06	0.49	−0.05	0.14

^***^Means *P*-value less than 0.001.

## 5 Discussion

### 5.1 Theoretical contributions

This paper makes three contributions in the theoretical aspect. Foremost, this study verifies the positive main effect of mortality salience on consumers’ green food purchase intention, expands the contextual application of Terror Management Theory (TMT) in the field of daily consumption decision-making. On the one hand, most existing TMT-based consumption studies focus on luxury consumption (59), pro-environmental behavior in general (60), but lack targeted empirical tests on the specific scenario of green food consumption, which integrates both personal health protection and environmental sustainability attributes. Through 4 experiments, this study consistently verifies that mortality salience can significantly and positively promote consumers’ green food purchase intention, confirming that green food can be used as an effective consumption carrier to alleviate death anxiety. On the other hand, previous TMT literature has mostly discussed the two core defensive systems as relatively independent or alternative response paths to death anxiety (6, 61). Our findings show that, when confronted with mortality salience, consumers do not make an either-or choice between egoistic health appeals and altruistic environmental appeals. Instead, they integrate these motives into a single existential continuum. Thus, under the experimental conditions and sample of this study, green consumption thus operates as a dual buffer against death anxiety, offering the experimentally replicable micro-mechanism bridging ecological psychology and existential psychology.

Second, this study clarifies the internal psychological mechanism of how mortality salience affects green food consumption decision-making, and provides an integrated theoretical explanation framework for the coexistence of approach and avoidance motivation in green consumption. We demonstrated that mortality salience can simultaneously activate two motivations within the same consumption context: (a) an avoidance motivation centered on protecting one’s own and family’s health, and (b) an approach motivation focused on enhancing social and environmental well-being. This finding not only complements the traditional assumption in prior research that approach and avoidance motivations compete, but also provides an empirical paradigm of “death-driven dual-motivation integration” for eco-existential psychology in the Chinese green food market. It reveals a psychological mechanism that green consumption can address the dual concerns of “reducing immediate threats” and “achieving future transcendence” among the study’s sample.

Furthermore, this study clarifies the differentiated moderating effects of perceived warmth and perceived competence on the dual mediating pathways, expands the contextual application of the Stereotype Content Model (SCM) in high-risk threat decision-making scenarios, and defines the boundary conditions of the dual-path model. Theoretically, this selective matching effect can be explained by two core mechanisms: first, motivational congruence, individuals in specific motivational states only process information consistent with their current goals. When mortality salience activates self-protective avoidance motivation, consumers prioritize competence cues that signal risk reduction; when it activates prosocial approach motivation, they only respond to warmth cues that signal collective care. Second, cognitive narrowing under threat, mortality salience depletes cognitive resources, making individuals unable to process multi-dimensional information simultaneously, thus selectively attending to the most goal-relevant cues. This finding clarifies the situational boundary of SCM and demonstrates that the effects of warmth and competence are not stable but depend on individuals’ motivational states.

Notably, the mediating effect of avoidance motivation was substantially stronger than that of approach motivation. Theoretically, this reflects the hierarchical nature of terror management defenses: self-esteem-based proximal defense, which addresses immediate survival threats, is evolutionarily prioritized over cultural worldview-based distal defense, which addresses long-term existential anxiety. This finding clarifies that self-protective motives dominate consumer responses to mortality salience in food consumption contexts.

In summary, this study supplements and perfects a three-tier hierarchical integrative framework, which clarifies the respective roles of TMT as the macro-level antecedent driver explaining motivational shift origins, AATM as the meso-level mediating mechanism translating abstract defenses into concrete behavioral tendencies, and SCM as the micro-level boundary condition specifying contextual moderators. This framework forms a complete explanatory chain from existential threat to consumption behavior, alleviates the theoretical fragmentation in previous studies, and offers a supplementary analytical reference for future research on threat-driven prosocial and pro-environmental consumption.

### 5.2 Practical implications

This research offers several practical insights seeking to promote green food consumption amid environmental change, public health emergencies, and food safety incidents that are applicable to similar consumer groups and contexts.

Foremost, during the “window period” of mortality salience triggered by public health or food safety crises, in addition to responding to the threats, governments and non-profit organizations may prioritize communications focused specifically on “green organic food” rather than broader green concepts. Specifically, they can launch two distinct food-specific information frames: “Zero Pesticides, Rigorous Testing, Protecting Your Family’s Dining Table” (to trigger avoidance motivation) and “Choose Organic Farming, Preserve Farmland and Clean Water for Future Generations” (to trigger approach motivation). In addition, they should collaborate with supermarkets and fresh food platforms to create “Safe Green Organic Food Zones,” could help the public to quickly translate green eating into immediate, tangible daily actions during crises, though real-world implementation requires further validation.

More importantly, for green food enterprises operating in a threat-laden environment, in addition to abiding by basic ethical constraints (using only objective risk information, refraining from creating or exaggerating mortality threats, protecting vulnerable groups such as women, children, the elderly and pregnant women from excessive fear appeals, and ensuring transparent and verifiable product claims) and conveying genuine empathy for consumers’ legitimate health and safety concerns, they can adopt differentiated marketing strategies and value propositions. Specifically, for family-oriented consumers prioritizing self and household protection, highlight competence cues including third-party safety test reports, traceable supply chains and rigorous quality control protocols to reinforce risk-avoidance motivations; for eco-conscious consumers seeking collective and intergenerational benefits, emphasize warmth cues such as small-scale farmer partnerships, regenerative agriculture practices and community-focused initiatives to amplify prosocial approach motivations. This solution-centered approach not only effectively translates consumers’ crisis-induced psychological needs into immediate green food purchase behaviors but also builds enduring brand trust and fosters long-term sustainable consumption habits beyond the crisis period.

In addition, for individuals facing pandemics or disasters, we recommend reframing the simple act of “buying green organic food” as a dual-purpose goal: immediate self-protection and long-term social contribution. While shopping, consciously scan packages for safety-test data (competence cue) alongside environmental stories or farm-of-origin narratives (warmth cue). This turns routine purchases into self-transcendent actions that alleviate death anxiety and heighten perceived meaning, making green consumption more likely to become a lasting habit with long-term behavioral verification needed.

### 5.3 Limitations and future research

First, prior evidence suggests that mortality salience activates two defensive mechanisms, namely self-esteem and cultural worldview (5, 7), and our preliminary hypotheses suggest that these mechanisms differentially drive approach and avoidance motivations. However, existing scales lack the specificity required to capture the distinct manifestations of self-esteem and cultural worldview within the green consumption context. Consequently, the internal mechanisms underlying these two pathways remain insufficiently delineated. Therefore, future research will develop tailored measures of self-esteem and cultural worldview specifically designed for green consumption settings, enabling a more nuanced examination of the separate mechanisms through which mortality salience influences green food purchase intention.

Second, this study has not fully resolved the ongoing replication problem of mortality salience effects in TMT research. Although we optimized the induction paradigm to improve the robustness of the core effect, our findings are still based on a single cultural context (Chinese consumers), specific threat-related induction materials, and laboratory experimental design. As demonstrated by Treger et al. (62), mortality salience effects are highly sensitive to cultural framing, sample characteristics, and experimental paradigms, meaning the generalizability of our dual-path model across different cultural backgrounds, real-world consumption scenarios, and alternative induction methods remains to be fully verified. Future research will adopt pre-registered experimental designs with large-scale, cross-cultural, and representative samples to systematically test the replicability of our core findings, incorporate more comprehensive manipulation check batteries to rule out potential confounding perceptual dimensions and verify the purity of competence and warmth manipulations, and further clarify the boundary conditions of mortality salience effects in green consumption contexts, to directly respond to the core challenges.

Third, the experimental scenarios for manipulating perceived warmth and competence have certain artificiality. To isolate the independent effects of the two dimensions and avoid confounding, we adopted an extreme manipulation approach that completely separated warmth and competence cues, whereas real-world green food brands typically convey both simultaneously with different emphases. We also used a fictitious brand and controlled laboratory setting to eliminate pre-existing brand biases. While our manipulation was rigorously validated in pre-tests, future research could use real brands and field experiments to improve ecological validity.

Finally, there remains room for further optimization in the sampling design to enhance the generalizability of our research findings. The sample is primarily concentrated in a single geographic region in central China. Given that consumer psychological responses to mortality salience and green food purchase decisions may vary across regions with different levels of economic development, cultural norms and environmental awareness, the cross-regional generalizability of our model can be further verified in subsequent studies. To address these aspects, future research can adopt a more diversified sampling design, recruit large-scale cross-regional and cross-cultural samples to further improve the external validity of the findings. Specifically, we will recruit participants through online subject recruitment platforms such as Amazon Mechanical Turk (MTurk) in subsequent studies to break the geographic limitations of offline recruitment.

## 6 Conclusion

This paper explored whether mortality salience boosts intention to purchase green food and clarified the dual-path process behind this effect. Results show that mortality salience positively predicts green food purchase intention, and this effect is jointly mediated by approach motivation focused on collective and environmental well-being and avoidance motivation centered on personal and family health protection. Moreover, warmth and competence perceptions of green food moderate the two pathways: high warmth amplifies the link between mortality salience and approach motivation, whereas high competence strengthens the link between mortality salience and avoidance motivation. These findings indicate that, during public-health crises, policymakers and marketers should tailor messages and product cues to simultaneously activate both motives, thereby encouraging immediate and sustained green food consumption.

These findings extend TMT, AATM, and SCM to the green food consumption domain, and provide empirical evidence for the integration of self-protective and altruistic motives under death-related threats. Practically, the results offer actionable guidance for policymakers, green food enterprises, and consumers to promote green consumption during crisis contexts with the caveat that these implications require validation in real-world settings and broader populations.

This research also enriches the understanding of consumer psychological responses to existential threats, and helps mitigate public death anxiety while boosting the green food market. Future work addressing the identified limitations will further refine the dual-path model and enhance its real-world applicability.

## Data Availability

The datasets presented in this study can be found in online repositories. The names of the repository/repositories and accession number(s) can be found in the article/supplementary material.
